# Establishment of m7G-related gene pair signature to predict overall survival in colorectal cancer

**DOI:** 10.3389/fgene.2022.981392

**Published:** 2022-10-14

**Authors:** Kai Li, Weixing Wang

**Affiliations:** ^1^ Department of Gastrointestinal Surgery Ⅱ, Renmin Hospital of Wuhan University, Wuhan, China; ^2^ Department of General Surgery, Renmin Hospital of Wuhan University, Wuhan, China

**Keywords:** 7-methylguanosine, colorectal cancer, prognosis, tumor microenvironment, treatment response

## Abstract

**Background:** N7-methylguanosine (m7G) is an emerging research hotspot in the field of RNA methylation, and its role in tumor regulation is becoming increasingly recognized. However, its role in colorectal cancer (CRC) remains unclear. Hence, our study explored the role of m7G in CRC.

**Methods:** The mRNA expression data and the corresponding clinical information of the patients with CRC were obtained from The Cancer Genome Atlas (TCGA). A m7G-related gene pair signature was established using the Cox and LASSO regression analyses. A series of *in silico* analyses based on the signature included analysis of prognosis, correlation analysis, immune-related analysis, and estimation of tumor mutational burden (TMB), microsatellite instability (MSI), and response to immunotherapy. A nomogram prediction model was then constructed.

**Results:** In total, 2156 m7G-related gene pairs were screened based on 152 m7G-related genes. Then, a prognostic signature of seven gene pairs was constructed, and the patients were stratified into high- or low-risk groups. Better overall survival (OS), left-sided tumor, early stage, immune activity, and low proportion of MSI-low and MSI-high were all associated with a low risk score. High-risk patients had a higher TMB, and patients with a high TMB had a poor OS. Furthermore, the risk score was linked to immune checkpoint expression (including PD-L1), the tumor immune dysfunction and exclusion (TIDE) score, and chemotherapy sensitivity. We also created an accurate nomogram to increase the clinical applicability of the risk score.

**Conclusion:** We identified an m7G pair-based prognostic signature associated with prognosis, immune landscape, immunotherapy, and chemotherapy in CRC. These findings could help us to better understand the role of m7G in CRC, as well as pave the path for novel methods to assess prognosis and design more effective individualized therapeutic strategies.

## Introduction

Colorectal cancer (CRC) is the third most common tumor and the second leading cause of cancer-related deaths worldwide, with an estimated 1.8 million new cases and approximately 915,880 deaths worldwide in 2020 (Sung et al., 2021). With increasing incidence and high mortality rates, CRC has become a serious threat to human health (Siegel et al., 2021). Although colonoscopy has provided a better screening method for diagnosing early CRC, many people cannot accept this kind of examination because of its price, psychological pressure, and related risks (Kanth and Inadomi, 2021). Most patients were diagnosed in the middle and late stages of the disease. Currently, CRC can be treated with surgery, chemotherapy, radiotherapy, and biotherapy. However, nearly 40% of CRC patients eventually experience tumor relapse or late metastasis, with less than 15% of patients surviving for more than 5 years (Basak et al., 2020). Consequently, it is important to identify new biomarkers that effectively determine the prognosis of CRC.

RNA methylation has been reported to be associated with various physiological processes and diseases, and abnormal methylation can lead to disease and cancer (Zhang et al., 2021a; Song et al., 2021). To date, more than 170 distinct RNA modifications have been identified (Frye et al., 2018). RNA modification plays an important role in regulating gene expression (Frye et al., 2018). Among these, RNA methylations such as N1-methyladenosine (m1A), N6-methyladenosine (m6A), 5-methylcytosine (m5C), and 7-methylguanosine (m7G) have a variety of biological properties (Shi et al., 2019; Shi et al., 2020; Zhang et al., 2021b; Wei and He, 2021). m6A is the most abundant mRNA modification in eukaryotic cells and is regulated by effector proteins, writers, readers, and erasers (Shi et al., 2019). m6A regulators have been reported to play crucial roles in various biological functions *in vivo* (He et al., 2019; An and Duan, 2022). m7G is the most prevalent modification in the 5’ cap of mRNA and is catalyzed by the Trm8/Trm82 complex in yeast and by the METTL1 andWDR4 complexes in humans under the action of methyltransferases (Alexandrov et al., 2002; Luo et al., 2022). m7G can be found not only in mRNA caps but also in several internal sites of mRNAs, tRNAs, and rRNAs (Pandolfini et al., 2019; Dai et al., 2021; Ma et al., 2021). Growing evidence suggests that m7G modification can regulate mRNA transcription, nuclear processing, tRNA stability, miRNA biological function, and maturation of 18S rRNA and plays a vital role in oncogenic mRNA translation and cancer development (Luo et al., 2022). The m7G regulators have been shown to be prognostic factors in multiple cancer types. METTL1 and WDR4 are upregulated in various types of cancers, which are associated with poor prognosis in patients with cancers such as esophageal squamous cell carcinoma (Han et al., 2022), intrahepatic cholangiocarcinoma (Dai et al., 2021), and nasopharyngeal carcinoma (Chen et al., 2022). Currently, most studies have only focused on the role of a single m7G regulator. However, tumorigenesis is determined by multiple genes, and the prognostic role of multiple m7G regulators has not yet been elucidated.

With the development of RNA sequencing and the establishment and improvement of large tumor databases such as The Cancer Genome Atlas (TCGA), it is possible to systematically study the role of m7G regulators in CRC. Therefore, we developed a novel prognostic signature based on seven m7G-related gene pairs. We systematically and comprehensively investigated the potential role of the model in the clinical outcomes and tumor microenvironment (TME) of CRC. Our results provide additional data on prognostic biomarkers and therapeutic targets for CRC.

## Materials and methods

### Data source and preprocessing

We collected the original gene expression profiling data of 571 CRC and 44 adjacent normal tissues and the clinical characteristics of CRC cohorts from the TCGA database (https://portal.gdc.cancer.gov/). Patients without prognostic data were excluded from this study. In addition, data related to 562 patients were obtained from the Gene Expression Omnibus (ID: GSE29582; http://www.ncbi.nlm. nih.gov/geo/) databases. The TCGA dataset (training set) was employed to develop a prognostic signature, and the GSE39582 dataset (validation set), which contained data on 403 CRC samples, was used to validate the predictive accuracy of the signature. Additionally, data regarding 152 m7G regulators were obtained from a previous study (Ming and Wang, 2022).

### Construction of m7G-related gene pairs

Pairwise comparisons of the overlapping m7G-related gene expression profiles derived from the training and validation sets were performed. Using the R software, we examined the data relating to m7G-related gene A and m7G-related gene B in each CRC sample to determine a score for each pair. The algorithm presents a scoring system in which the score of the m7G-related gene pair is 1 if the expression level of the first m7G-related gene is higher than that of the second m7G-related gene; otherwise, it is 0, resulting in the construction of a 0-or-1 matrix. An m7G-related gene pair was deemed invalid if its proportion was <20% or >80% of the samples in either the training or validation sets, respectively (Heinäniemi et al., 2013). Following the screening, the remaining pairs were used for subsequent investigations.

### Establishment and validation of the prognostic signature based on m7G-related gene pairs

To discover the OS-related m7G-related gene pairs, a univariate Cox regression analysis was conducted based on the m7G-related gene pairs in the training set. A threshold of p-value < 0.05 was set to screen the prognostic variables. LASSO penalized Cox proportional hazards regression was used to further minimize the risk of over-fitting based on m7G prognostic gene pairs. Subsequently, multivariate Cox analysis was used to select the candidate gene pairs required to establish a prognostic signature. Finally, a signature, termed the risk score, was constructed using seven m7G-related gene pairs and their correlative coefficients acquired in the training set. The risk score for each patient was calculated as follows:

Risk score= (gene pair A coefficient × expression level) + (gene pair B coefficient × expression level) +…+ (gene pair n coefficient × expression level). Furthermore, according to the above formula, the risk scores of patients with CRC were calculated separately, and the patients were stratified into low- and high-risk groups based on the median risk score. Kaplan–Meier survival curves were plotted to estimate the OS differences between the two subgroups. A time-dependent ROC (ROC) curve was plotted to assess the accuracy of the signature.

The external cohort GSE39582 was used to verify the m7G-related prognostic model. The risk score for each patient was calculated using the same coefficients and normalized expression microarray data for the m7G pairs. The patients were stratified into low- and high-risk groups based on the median risk score of the training set. Kaplan-Meier and tdROC curves were plotted to evaluate the prognostic value of the risk model.

### The prognostic value of the risk score

The associations between the risk score and clinicopathological traits (age, sex, tumor site, stage, and tumor status) were compared. Univariate and multivariate analyses were performed to evaluate whether the risk scores were independent of the other clinical variables. Moreover, we conducted a stratified analysis to determine whether the risk score preserved its predictive capacity in the different subgroups.

### Establishment and validation of a nomogram scoring system

Based on the independent indicators, the R software “regplot” package was used to develop a nomogram for OS prediction at three and 5 years. Then, tdROC analysis for 3- and 5-year OS was performed to evaluate the prognostic accuracy, and the calibration curves were drawn to compare nomogram-predicted probability with actual survival.

### Immune-related analysis of colorectal cancer patients using the prognostic signature

The fraction of immune cells in the two risk subgroups was assessed using the CIBERSORT algorithm. Differences between the low- and high-risk groups were detected using the Wilcoxon rank-sum test. We also used boxplots to evaluate the differences in the levels of expression of immunological checkpoints between the low- and high-score groups, which were retrieved from the literature. Moreover, we calculated the tumor immune dysfunction and exclusion (TIDE) score to predict the response to immunotherapy in CRC patients (Jiang et al., 2018).

### Tumor mutation burden and microsatellite instability analyses

The “maftools” R package was employed to reveal the mutation frequency in patients with CRC belonging to different risk subgroups. Subsequently, we analyzed the relationship between TMB and the risk scores. Using the Kaplan–Meier method, the OS rates derived from data pertaining to the CRC samples from the low- and high-TMB groups were compared. Next, we evaluated the synergistic effect of TMB and risk score on prognostic stratification. In addition, the association between the risk scores and MSI was explored.

### Potential drugs for patients with colorectal cancer

We explored the sensitivity of chemotherapy based on the gene expression levels using the “pRRophetic” R package (Geeleher et al., 2014). The chemotherapeutic response was assessed based on the half-maximal inhibitory concentration (IC50) of each sample.

### Functional enrichment analysis

The differentially expressed genes (DEGs) were screened using the “limma” package in the R software by setting criteria with |log2 (fold change, FC) |> 1 and adjusted p-value < 0.05. Subsequently, the “Clusterfiler” R package was employed to perform the Gene Ontology (GO) and Kyoto Encyclopedia of Genes and Genomes (KEGG) pathways.

### Statistical analysis

All analyses in this study were performed using the R software (version 4.0.3). Statistical significance was set at *p* < 0.05 unless otherwise stated.

## Results

### Establishment and validation of the prognostic signature based on m7G-related gene pairs

**FIGURE 1:**
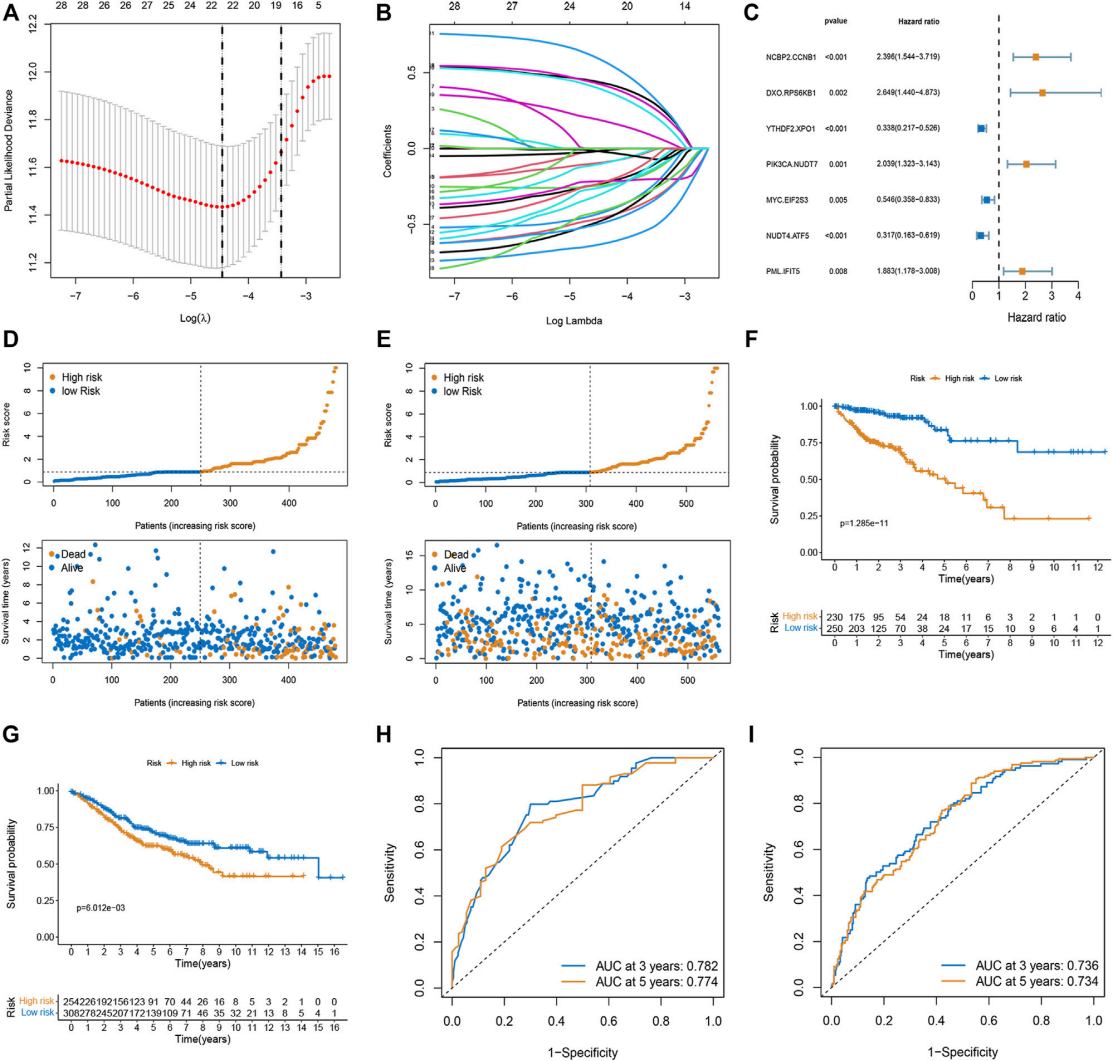
Construction and validation of the prognostic model based on m7G-related gene pairs. **(A)** Plot indicating the optimal λ selection by 10 cross-validated partial likelihood deviance of the LASSO-Cox regression. 10-fold cross-validated partial likelihood deviance was plotted against log(λ). **(B)** Plot of estimated coefficients from the LASSO-Cox regression against log(λ). Finally, 22 non-zero m7G pairs were selected. **(C)** Constructing a stepwise Cox proportional hazards model. **(D,E)** The distribution of each patient’s risk score ordered from low to high in training and validation sets. Patients are divided into two risk score level groups. Scatter diagram of the OS-time against the patients’ rank of risk score in training and testing sets. **(F,G)** Overall survival curves of different risk subgroups in the training and validation sets. **(H,I)** AUC curves to predict the sensitivity and specificity of 3- and 5-year survival according to the risk score in training and validation sets.

The m7G-related genes in the TCGA and GEO datasets were merged into an overlapping gene set, from which 135 m7G-related genes were shared in both sets. Pairwise comparisons were conducted using the algorithm described in the “Methods” section to calculate the risk score for each m7G-related gene pair for subsequent analysis. As a result, 2,156 m7G-related gene pairs were identified. We conducted a univariate Cox regression analysis on these pairs and selected 30 meaningful m7G pairs to generate the models (Supplementary Figure S1). Next, LASSO Cox regression analysis yielded 22 m7G pairs (Figures 1A,B), which were subsequently used to perform multivariate Cox regression analysis. Based on the Akaike information criterion value, we obtained seven m7G pairs to construct risk models, including the NCBP2.CCNB1, DXO. RPS6KB1, YTHDF2. XPO1, PIK3CA.NUDT7, MYC. EIF2S3, NUDT4. ATF5, and PML. IFIT5 gene pairs (Figure 1C). The correlation coefficients are listed in Table 1. A risk score in the training set was assigned to each patient based on the expression levels of the seven m7G pairs multiplied by the regression coefficients obtained from multivariate Cox regression analysis.

**TABLE 1:** Multivariate Cox regression analysis of 11 m7G pairs associated with overall survival in patients with CRC.

id	Coef	HR	HR.95L	HR.95H	p-value
NCBP2.CCNB1	0.873933	2.396317	1.543864	3.719457	<0.001
DXO.RPS6KB1	0.974044	2.648634	1.439655	4.872875	0.001
YTHDF2.XPO1	−1.08373	0.338332	0.217453	0.526404	<0.001
PIK3CA.NUDT7	0.712567	2.039219	1.322867	3.143484	0.001
MYC.EIF2S3	−0.60472	0.546229	0.358296	0.832738	0.004
NUDT4.ATF5	−1.14792	0.317297	0.162668	0.618914	<0.001
PML.IFIT5	0.632646	1.882586	1.178223	3.008028	0.008

Risk score= (0.873933 × NCBP2.CCNB1 exp) + (0.974044 × DXO. RPS6KB1 exp) + (−1.08373 × YTHDF2. XPO1 exp) + (0.712567 × PIK3CA.NUDT7 exp) + (−0.60472 × MYC. EIF2S3 exp) + (−1.14792 × NUDT4. ATF5 exp) + (0.632646 × PML. IFIT5 exp).

We calculated the risk scores of each sample in the training set and classified the patients into two subgroups (low- and high-risk). In both the training and validation sets, the risk distribution plot demonstrated that survival times increased with the risk score (Figures 1D,E). The Kaplan-Meier curve indicated that low-risk patients had a more favorable survival time than high-risk patients in both the training and validation sets (Figures 1F,G). The AUC values for three and 5 years in the training set were 0.782 and 0.774, and the AUC values for three and 5 years in the validation set were 0.736 and 0.734 (Figures 1H,I).

### Clinical correlation analysis and stratification analysis of the risk score

**FIGURE 2:**
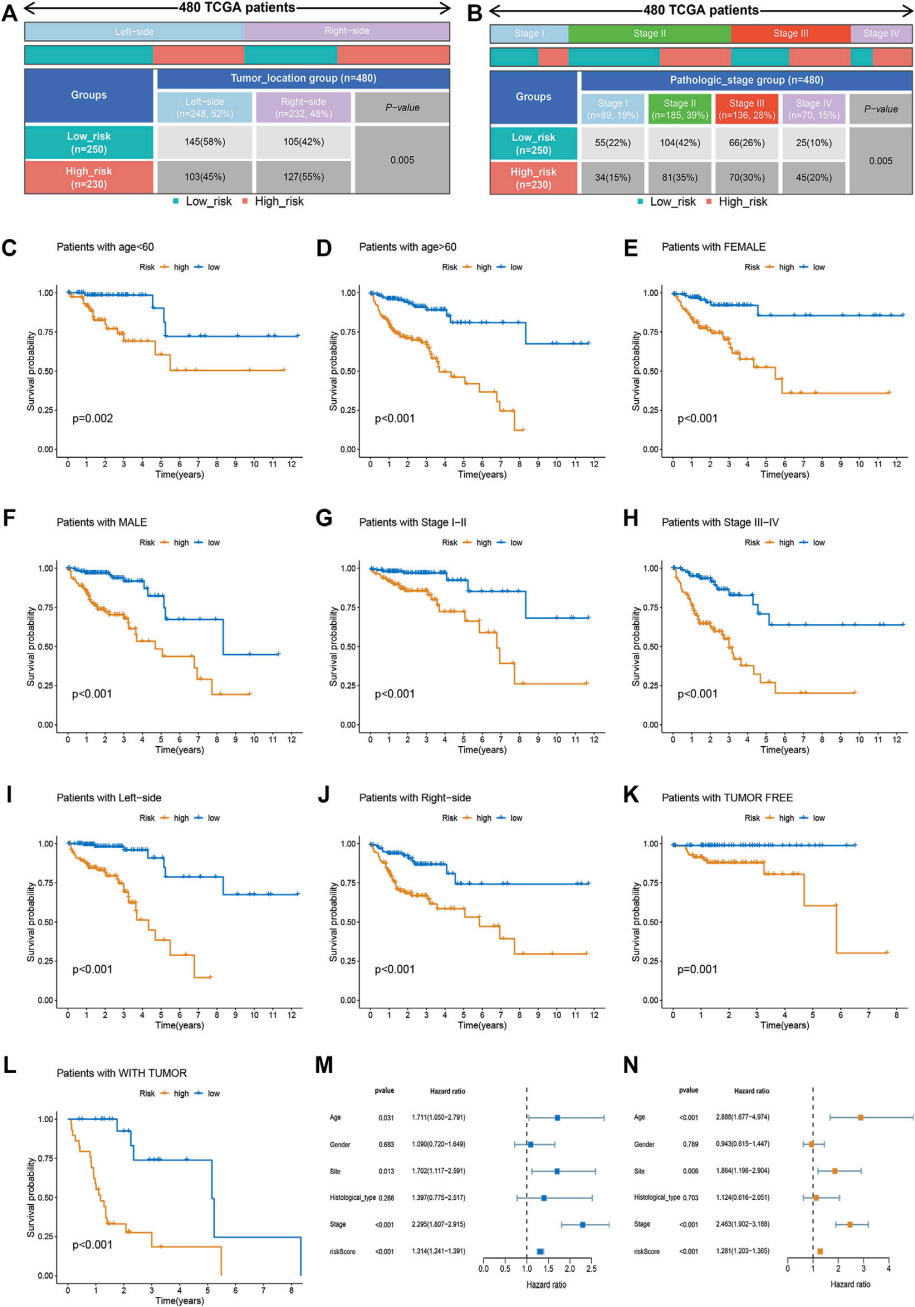
Clinical correlation analysis and stratification analysis of the risk score. **(A,B)** The relationship between the risk score and tumor site and TNM stage. **(C–L)** The Kaplan-Meier survival curves were stratified by different clinicopathological variants, including age, sex, tumor site, TNM stage, and tumor status. **(M)** The clinicopathological traits and risk score were analyzed by univariate Cox regression with the OS. **(N)** Multivariate Cox regression analysis revealing clinicopathological traits and risk scores related to OS.

The relationship between risk score and clinicopathological factors was explored. We found that patients with right-sided CRC and advanced stage (stage III-IV) were associated with high-risk scores, whereas sex and tumor status showed no correlation with risk scores (Figures 2A,B). Further survival analysis revealed that the risk scores could accurately predict the prognosis of patients with all the stratified clinicopathological variants (all *p* < 0.05, Figures 2C–L). Furthermore, we incorporated the risk scores into the corresponding clinical characteristics. Our results from univariate and multivariable Cox regression analyses showed a strong correlation between the risk scores and the prognosis in patients with CRC (Figures 2M,N).

### Generation and validation of a nomogram scoring system

**FIGURE 3:**
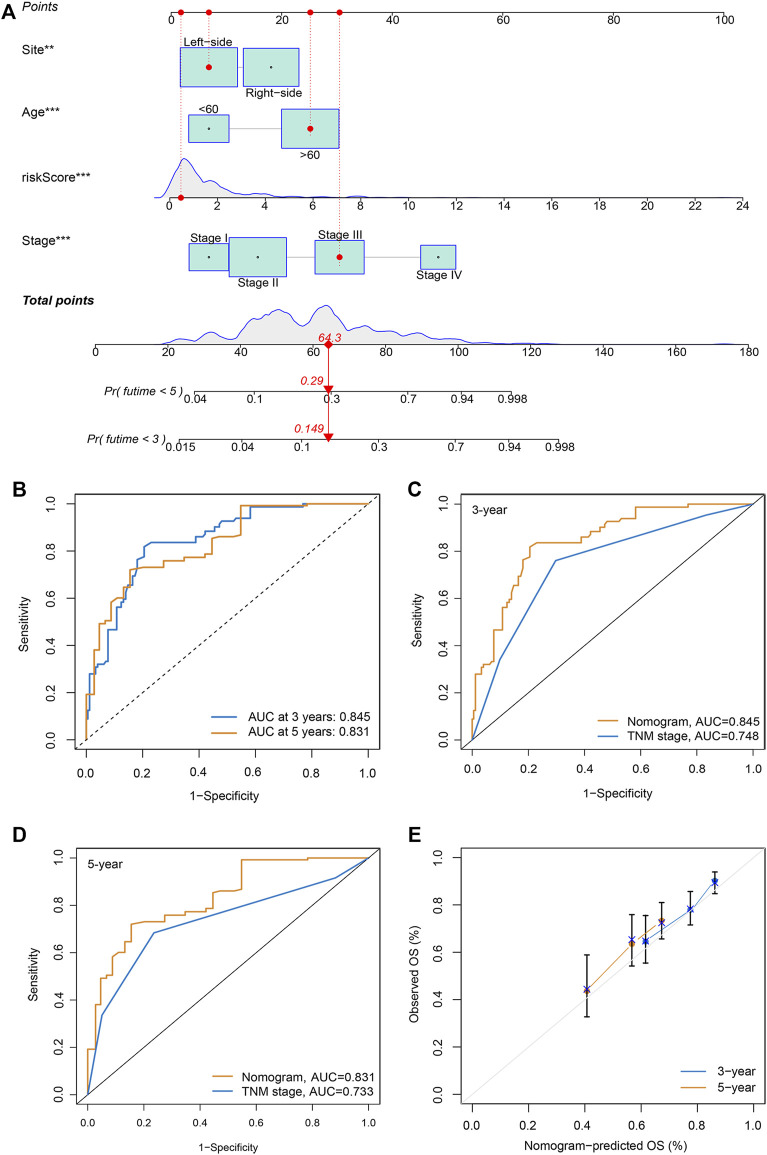
Generation and validation of a nomogram scoring system. **(A)** A nomogram for both clinicopathological traits and risk score to predict 3-year and 5-year OS. **(B)** The 3- and 5-year AUC values to evaluate the performance of the nomogram. **(C,D)** Comparison of the AUCs of the nomogram and TNM staging system at 3 and 5 years. **(E)** Calibration curve of the nomogram for predicting 3- and 5-year OS.

To generate personalized predictions for patients with CRC, we integrated the m7G score and clinicopathological parameters (age, tumor site, and stage) to establish a nomogram scoring system (Figure 3A). The predictive accuracy of the nomogram was assessed using receiver operating characteristics and calibration curves. Figure 3B shows that the 3- and 5-year AUC values of the nomogram were 0.845 and 0.831, respectively. The AUC values of the nomogram at years 3 and 5 were all greater than the AUC values for the TNM stage (Figures 3C,D). Lastly, results from the calibration curve analyses showed that the calibration curve of prognostication was close to the standard curve at the 3- and 5-year follow-ups, which again confirmed the inference above (Figure 3E).

### Evaluation of tumor microenvironment and immunotherapy response

**FIGURE 4:**
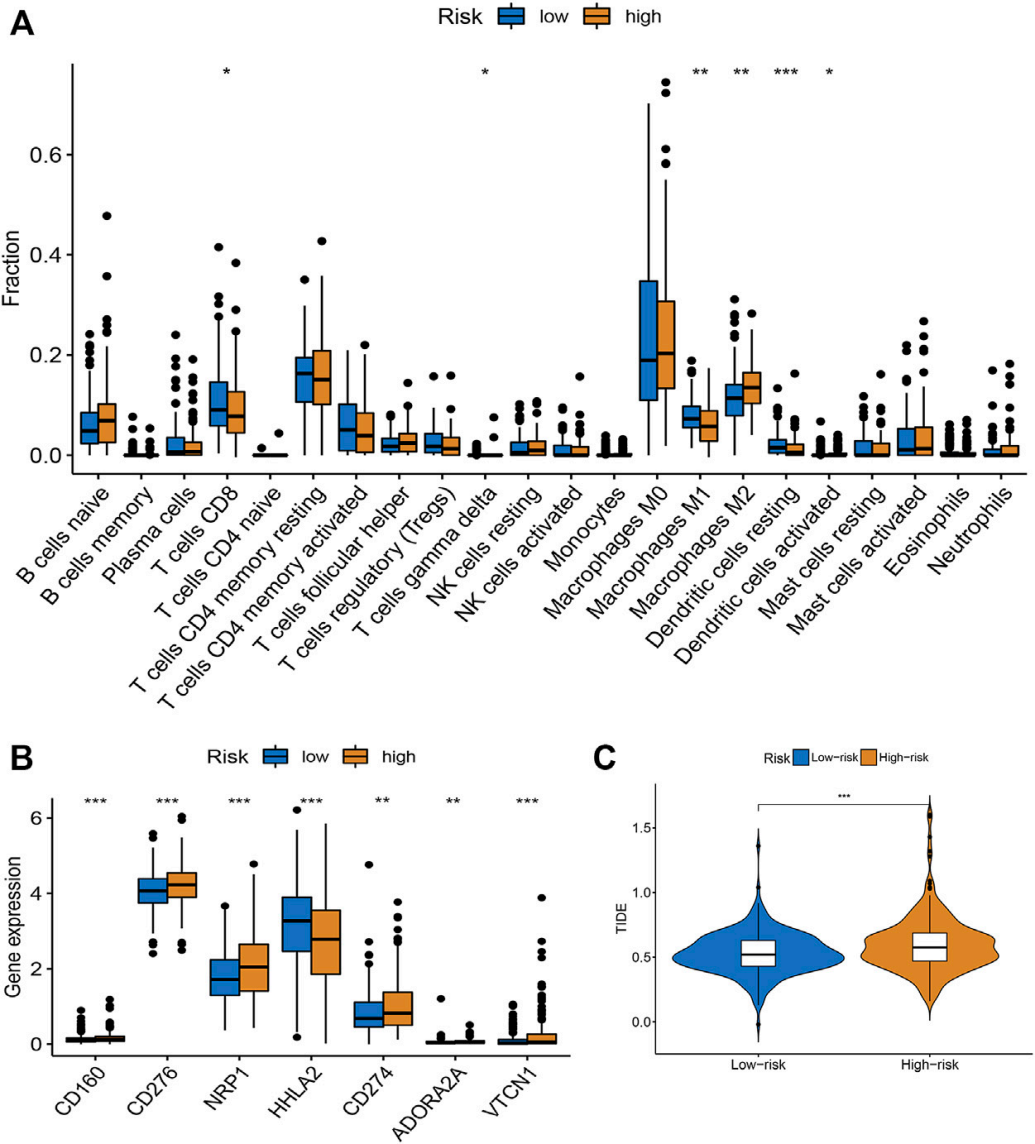
Evaluation of TME and immunotherapy response in different subgroups. **(A)** Comparison of 22 immune cells between low- and high-risk groups. Median values and IQR for each cell subset were calculated for each patient group and compared between the two groups using the Wilcoxon rank sum test. **(B)** Expression of common immune checkpoints in high- and low-risk groups. **(C)** The difference of stromal score, immune score, and ESTIMATE score between high- and low-risk groups. *p < 0.05, **p < 0.01, ***p < 0.001.

Based on the CIBERSORT algorithm, we noticed a decreased number of activated CD8 + T cells, M1 macrophages, and dendritic cells, as well as resting dendritic cells, and an increased number of M2 macrophages in the high-risk subgroup (Figure 4A). The expression levels of immune checkpoint-related genes in the low- and high-risk subgroups were investigated. We observed that PD-L1 (CD274), CD160, CD276, NRP1, HHLA2, ADORA2A, and VTCN1 levels were increased in the high-risk subgroup (Figure 4B). Moreover, the TIDE algorithm was employed to predict the patient’s response to immunotherapy. As illustrated in Figure 4C, higher TIDE scores were observed in the high-risk group than in the low-risk group, indicating that the patients were more prone to immune escape (Figure 4C). Taken together, the risk score may serve as a potential biomarker for predicting the response of patients with CRC to immunotherapy.

### Relationship between risk score and tumor mutational burden and microsatellite instability in colorectal cancer

**FIGURE 5:**
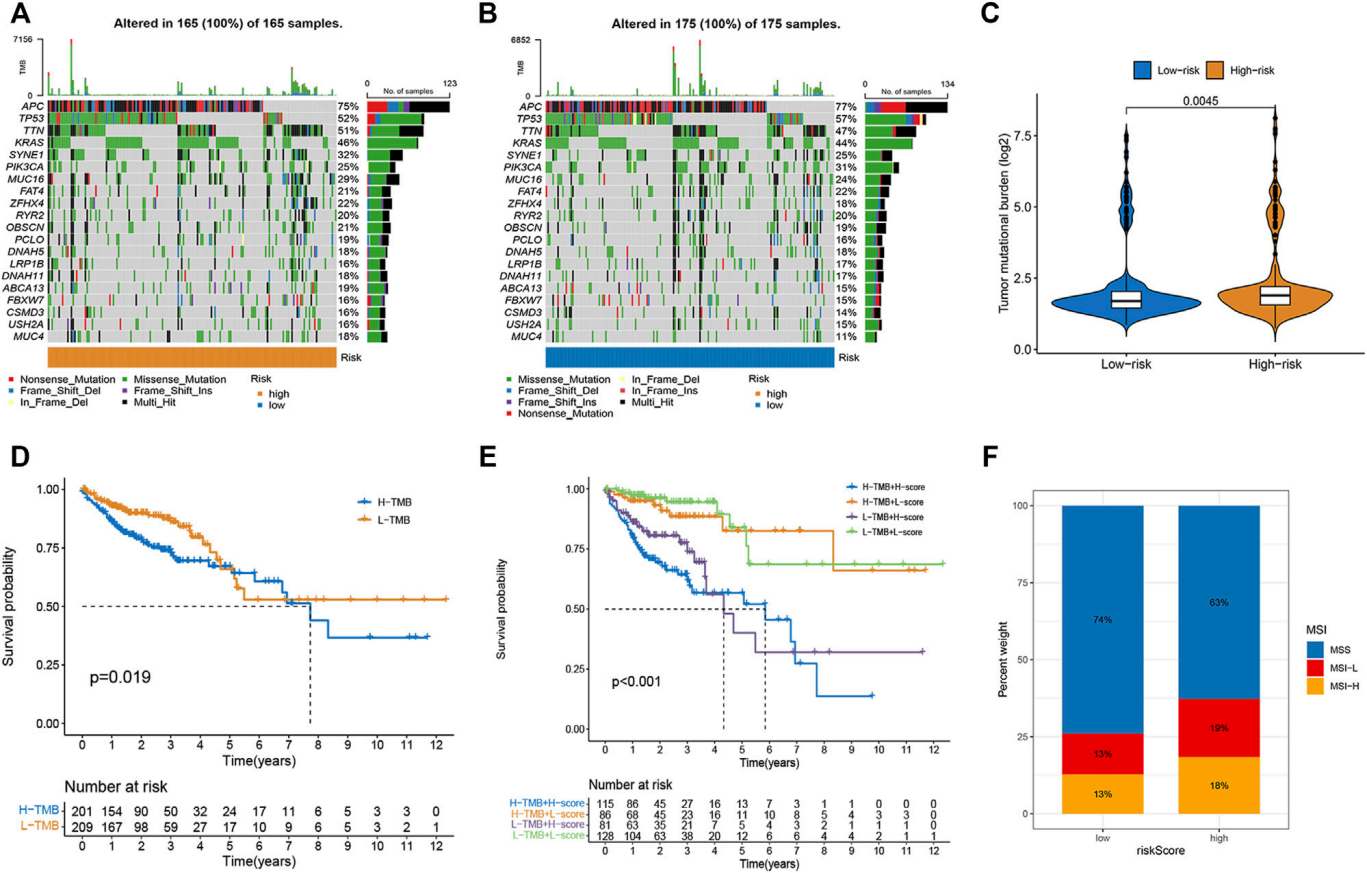
Correlations of risk score with TMB and MSI in CRC. **(A,B)** The mutation rate and types of top 15 genes in low- and high-risk groups. **(C)** The difference in TMB between high- and low-risk group. **(D)** Kaplan-Meier curve to show survival of patients in different TMB groups. **(E)** Kaplan-Meier survival curves to show the survival of patients in different risk scores and TMB groups. **(F)** Differences in the proportion of different microsatellite statuses between two risk groups.

As illustrated in the waterfall charts, the mutated genes in the different risk groups were mainly APC, PIK3CA, SYNE1, KRAS, TTN, TP53, and MUC16, but the mutation rates of these genes were different in the high- and low-risk subgroups (Figures 5A,B). TMB was higher in the high-risk subgroup (*p* = 0.004; Figure 5C), indicating that high-risk patients benefited more from immunotherapy. The Kaplan-Meier survival curve showed that the OS rate was higher in the low TMB group (*p* = 0.019; Figure 5D). A combined analysis of TMB and risk score demonstrated that the risk score was a prognostic factor independent of TMB (*p* < 0.001; Figure 5E). Moreover, higher proportions of MSI-H and MSI-L were observed in the high-risk group than in the low-risk group (Figure 5F).

### Exploration of chemotherapy sensitivity

**FIGURE 6:**
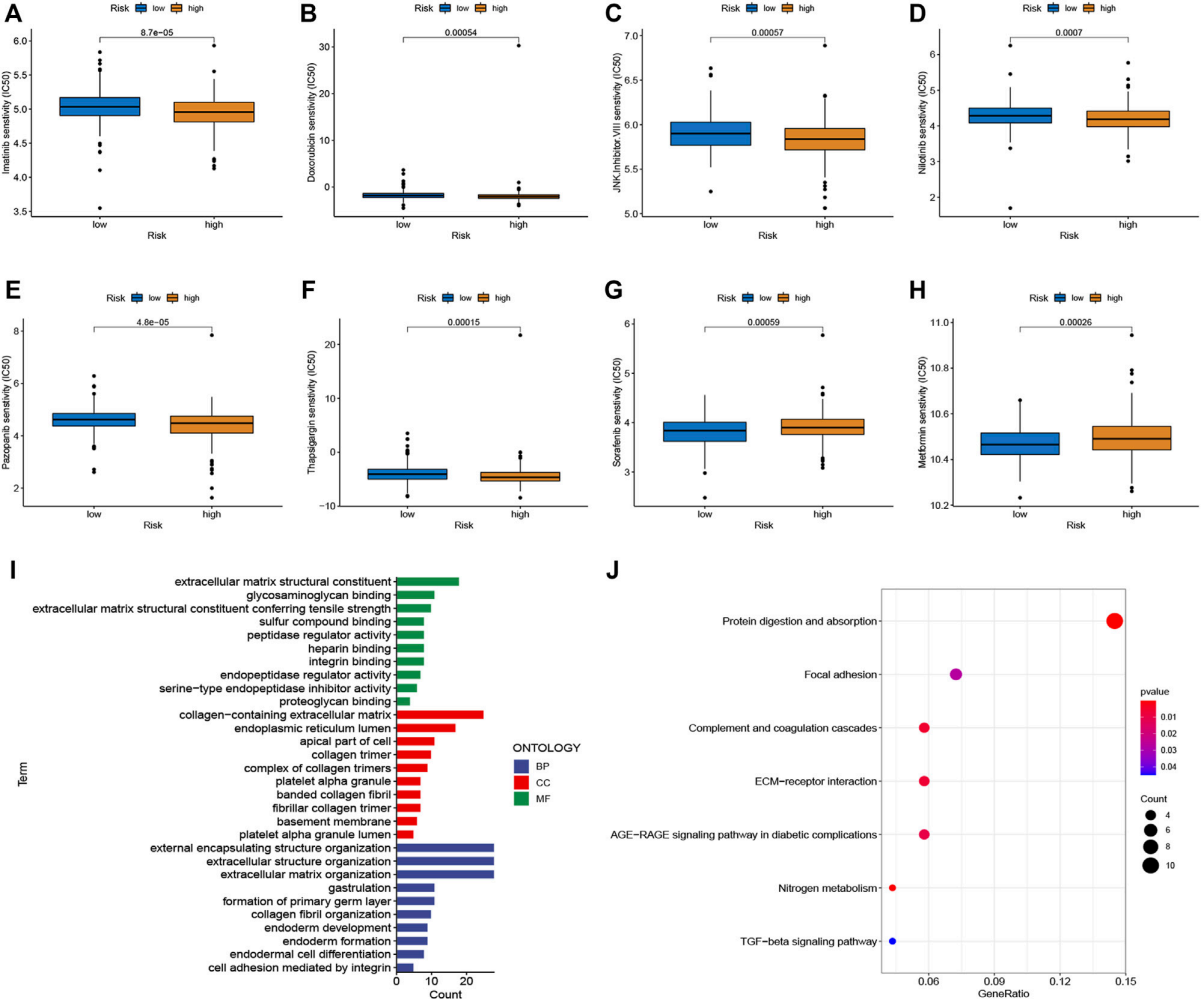
Chemotherapy sensitivity and functional enrichment analyses. **(A–H)** Boxplots of the IC50 values of the Imatinib, Doxorubicin, JNK Inhibitor VIII, Nilotinib, Pazopanib, Thapsigargin, Sorafenib, and Metformin between different risk subgroups. **(I)** Top enriched gene pathways/functions using GO terms of biological process, cellular component, and molecular function. **(J)** KEGG pathway analysis of DEGs between two risk groups. The bigger bubble represents more genes enriched, and the redder color means more obvious differences.

To select potential drugs for patients with CRC, we investigated the correlation between risk scores and drug sensitivity using the “pRRophetic” R package. The IC50 values of doxorubicin, imatinib, JNK Inhibitor VIII, nilotinib, pazopanib, and thapsigargin in the high-risk subgroup were lower, whereas sorafenib and metformin had lower IC50 values in the low-risk subgroup (Figures 6A–H). Taken together, the patients with CRC and high risk scores were more sensitive to doxorubicin, imatinib, JNK inhibitor VIII, nilotinib, pazopanib, and thapsigargin than low risk patients. In contrast, the patients with CRC and low risk scores were more sensitive to sorafenib and metformin.

### GO and KEGG pathway analysis

We performed functional enrichment analyses to investigate the latent biological processes that affect risk scores. Based on the criteria of FC > 1.5 and adjusted p-value < 0.05, we screened 262 DEGs in the different risk groups. GO terms indicated enrichment in the collagen-containing extracellular matrix, extracellular matrix structural constituents, and external encapsulating structure organization (Figure 6I). Furthermore, the results of the KEGG pathway analysis, as shown in Figure 6J, suggest that these DEGs were enriched in ECM-receptor interactions, protein digestion, absorption, complement and coagulation cascades, and focal adhesion.

## Discussion

CRC is a highly heterogeneous disease, and the difference in heterogeneity provides a complex landscape for the prognosis of patients and their response to immunotherapy. The traditional prognostic evaluation system based on TNM staging has not been able to meet the requirements of precision medicine. Exploring the molecular mechanisms underlying CRC pathogenesis may provide key clues for identifying promising prognostic biomarkers or developing effective therapeutic targets. A growing number of studies have established that post-transcriptional RNA modifications play a significant role in modulating gene expression, as well as carcinogenesis and development, among which the most common modifications are m6a, m5c, m1a, and m7G (Barbieri and Kouzarides, 2020; Haruehanroengra et al., 2020). Accumulating evidence suggests that m7G modifications actively participate in biological and pathological processes by affecting the metabolism of various RNA molecules. Aberrant m7G levels facilitate tumorigenesis and its progression by regulating multiple oncogenes and tumor suppressor genes (Luo et al., 2022). However, to the best of our knowledge, few studies have examined the role of m7G regulators and m7G pair-based signatures in CRC as prognostic biomarkers and therapeutic targets. Therefore, the construction of an m7G pair-based signature can not only predict the survival rate of patients but also provide deeper insights into the m7G-mediated immune response and chemoresistance, which will provide novel ideas and methods for determining the pathogenesis and effective treatment modalities for CRC.

In the present study, we developed a novel prognostic signature based on seven m7G gene pairs in patients with CRC using previously identified m7G-related genes with either higher or lower expression levels. Several m7G-related genes have been reported to be associated with CRC and other cancers. MYC is a master transcription regulator and is one of the most common oncoproteins associated with increased mortality (Castell and Larsson, 2015). MYC activates or represses the transcription of numerous genes involved in cellular processes (Meyer and Penn, 2008). MYC dysregulation occurs in most cancers and is often associated with aggressive disease, treatment resistance, and poor prognosis (Castell and Larsson, 2015). Schmidt et al. (Schmidt et al., 2019) revealed that the MYC-GCN2-eIF2α negative feedback loop restricts protein synthesis to prevent MYC-dependent apoptosis in CRC (Schmidt et al., 2019). Wu et al. (2021) indicated that MYC could bind to the promoter of the MNX1-AS1 locus and activate its transcription, further promoting CRC progression by stabilizing YB1. XPO1 is a nuclear export protein involved in cancer progression and therapy. Aladhraei et al. (2019) observed that XPO1 expression was elevated in CRC and that XPO1 overexpression was significantly associated with moderately/poorly differentiated tumors and advanced tumor stages. *In vitro* experiments showed that KPT-330, an XPO1 inhibitor, inhibited cancer growth in a dose- and time-dependent manner. Similarly, XPO1 inhibition has been shown to enhance therapeutic response in CRC (Ferreiro-Neira et al., 2016; Inoue et al., 2021). PIK3CA, the gene encoding the alpha catalytic subunit of PI3K, is dysregulated in multiple cancers (Voutsadakis, 2021). It is one of the most frequently mutated oncogenes in CRC. Its mutations are associated with higher gene mutation rates in other important cancer-related pathways, such as the Wnt/β-catenin and tyrosine kinase receptor/K-Ras/BRAF/MAPK pathways (Voutsadakis, 2021). Additionally, PIK3CA mutations have been reported to be associated with clinicopathological characteristics and prognosis (Mei et al., 2016; Jin et al., 2020).

Infiltrating immune cells are the primary cells in tumor tissues and play vital roles in tumor biology (Hinshaw and Shevde, 2019; Mao et al., 2021). This study investigated the immune status of different risk score groups. The infiltration of immune cells, such as activated CD8^+^ T cell M1 macrophages, activated dendritic cells activated, and resting dendritic cells, in the low-risk group was obviously lower than that of the high-risk subgroup, while the infiltration level of M2 macrophages was increased in the high-risk score group. CD8^+^ T cell infiltration levels are usually associated with favorable clinical outcomes in most solid tumors (van der Leun et al., 2020). The low-risk group was characterized by immune-mediated inflammatory activity, whereas the high-risk group was characterized by immunosuppression. Based on these findings, the worse survival outcomes in high-risk patients may have been caused by the decreased levels of antitumor immunity. Immunotherapy has provided a new perspective on tumor treatment (Riley et al., 2019; Zhang and Zhang, 2020). We assessed the response to immunotherapy in the high- and low-risk groups using the TIDE algorithm (Jiang et al., 2018), and the results showed that the high-risk group had higher TIDE scores, suggesting a poorer patient response to immunotherapy. Furthermore, we also evaluated the expression of immune checkpoint-related genes and found that PD-L1 (CD274), CD160, CD276, NRP1, HHLA2, ADORA2A, and VTCN1 expression increased in the high-risk score group compared to the low-risk score group, which means that these patients can benefit from ICIs. TMB is a complementary independent biomarker that can predict the efficacy of ICIs (Xiao et al., 2021). In this study, we found that the high-risk group had higher TMB and worse OS. Moreover, the risk score was found to be a prognostic factor independent of TMB. MSI has been used to classify different CRC subtypes (Chen et al., 2018). It is now well-established that patients with MSI-H tend to be more sensitive to ICIs (Lizardo et al., 2020). We found that high-risk scores were associated with MSI-H status in patients with CRC. Research on the sensitivity to chemotherapeutic drugs has always been a hot topic for scientists. We observed that high-risk patients were more sensitive to doxorubicin, imatinib, nilotinib, pazopanib, and thapsigargin, whereas low-risk patients were more sensitive to sorafenib and metformin. Taken together, the m7G pair signature holds promise for predicting the response to immunotherapy and targeted therapy, and it also provides new ideas for the selection of appropriate chemotherapeutic drugs.

Nevertheless, this study has some limitations. First, the signature was conducted based on public databases, which may have been influenced by inherent case selection bias. Second, clinical tissues and CRC cell lines should be employed to validate the expression levels of signature genes, and further investigation and experiments are needed to explore the detailed regulatory effects of m7G-related genes in CRC.

We established a novel m7G pair-based risk signature based on seven m7G-related pairs in CRC. The risk score is closely related to the clinical features and prognosis of patients. The nomogram can serve as a counseling and clinical decision aid for clinicians. Tumor microenvironment analysis established a theoretical framework for future research on the connection between immunity and m7G-related genes in CRC.

## Data Availability

Publicly available datasets were analyzed in this study. This data can be found here: The public datasets were obtained from TCGA (https://portal.gdc.cancer.gov/) and GEO (https://www.ncbi.nlm.nih.gov/geo/).

## Supplementary material

SUPPLEMENTARY FIGURE S1 Univariate Cox regression analysis of the variables affecting the prognosis of CRC patients.

## Abbreviations

CRC, colorectal cancer; m7G, N7-methylguanosine; MSI, microsatellite instability; TMB, tumor mutational burden; OS, overall survival; TIDE, tumor immune dysfunction and exclusion; TME, tumor microenvironment; DEGs, differentially expressed genes.

## References

[B1] Aladhraei M. Kassem Al-Thobhani A. Poungvarin N. Suwannalert P. (2019). Association of XPO1 overexpression with NF-κB and Ki67 in colorectal cancer. Asian pac. J. Cancer Prev. 20, 3747–3754. 10.31557/apjcp.2019.20.12.3747 31870117 PMC7173379

[B2] Alexandrov A. Martzen M. R. Phizicky E. M. (2002). Two proteins that form a complex are required for 7-methylguanosine modification of yeast tRNA. Rna 8, 1253–1266. 10.1017/s1355838202024019 12403464 PMC1370335

[B3] An Y. Duan H. (2022). The role of m6A RNA methylation in cancer metabolism. Mol. Cancer 21, 14. 10.1186/s12943-022-01500-4 35022030 PMC8753874

[B4] Barbieri I. Kouzarides T. (2020). Role of RNA modifications in cancer. Nat. Rev. Cancer 20, 303–322. 10.1038/s41568-020-0253-2 32300195

[B5] Basak D. Uddin M. N. Hancock J. (2020). The role of oxidative stress and its counteractive utility in colorectal cancer (CRC). Cancers (Basel) 12, E3336. 10.3390/cancers12113336 33187272 PMC7698080

[B6] Castell A. Larsson L. G. (2015). Targeting MYC translation in colorectal cancer. Cancer Discov. 5, 701–703. 10.1158/2159-8290.Cd-15-0660 26152922

[B7] Chen B. Jiang W. Huang Y. Zhang J. Yu P. Wu L. (2022). N(7)-methylguanosine tRNA modification promotes tumorigenesis and chemoresistance through WNT/β-catenin pathway in nasopharyngeal carcinoma. Oncogene 41, 2239–2253. 10.1038/s41388-022-02250-9 35217794

[B8] Chen L. Pan X. Hu X. Zhang Y. H. Wang S. Huang T. (2018). Gene expression differences among different MSI statuses in colorectal cancer. Int. J. Cancer 143, 1731–1740. 10.1002/ijc.31554 29696646

[B9] Dai Z. Liu H. Liao J. Huang C. Ren X. Zhu W. (2021). N(7)-Methylguanosine tRNA modification enhances oncogenic mRNA translation and promotes intrahepatic cholangiocarcinoma progression. Mol. Cell 81, 3339–3355.e8. 10.1016/j.molcel.2021.07.003 34352206

[B10] Ferreiro-Neira I. Torres N. E. Liesenfeld L. F. Chan C. H. Penson T. Landesman Y. (2016). XPO1 inhibition enhances radiation response in preclinical models of rectal cancer. Clin. Cancer Res. 22, 1663–1673. 10.1158/1078-0432.Ccr-15-0978 26603256

[B11] Frye M. Harada B. T. Behm M. He C. (2018). RNA modifications modulate gene expression during development. Science 361, 1346–1349. 10.1126/science.aau1646 30262497 PMC6436390

[B12] Geeleher P. Cox N. Huang R. S. (2014). pRRophetic: an R package for prediction of clinical chemotherapeutic response from tumor gene expression levels. PLoS One 9, e107468. 10.1371/journal.pone.0107468 25229481 PMC4167990

[B13] Han H. Yang C. Ma J. Zhang S. Zheng S. Ling R. (2022). N(7)-methylguanosine tRNA modification promotes esophageal squamous cell carcinoma tumorigenesis via the RPTOR/ULK1/autophagy axis. Nat. Commun. 13, 1478. 10.1038/s41467-022-29125-7 35304469 PMC8933395

[B14] Haruehanroengra P. Zheng Y. Y. Zhou Y. Huang Y. Sheng J. (2020). RNA modifications and cancer. RNA Biol. 17, 1560–1575. 10.1080/15476286.2020.1722449 31994439 PMC7567502

[B15] He L. Li H. Wu A. Peng Y. Shu G. Yin G. (2019). Functions of N6-methyladenosine and its role in cancer. Mol. Cancer 18, 176. 10.1186/s12943-019-1109-9 31801551 PMC6892141

[B16] Heinäniemi M. Nykter M. Kramer R. Wienecke-Baldacchino A. Sinkkonen L. Zhou J. X. (2013). Gene-pair expression signatures reveal lineage control. Nat. Methods 10, 577–583. 10.1038/nmeth.2445 23603899 PMC4131748

[B17] Hinshaw D. C. Shevde L. A. (2019). The tumor microenvironment innately modulates cancer progression. Cancer Res. 79, 4557–4566. 10.1158/0008-5472.Can-18-3962 31350295 PMC6744958

[B18] Inoue A. Robinson F. S. Minelli R. Tomihara H. Rizi B. S. Rose J. L. (2021). Sequential administration of XPO1 and ATR inhibitors enhances therapeutic response in TP53-mutated colorectal cancer. Gastroenterology 161, 196–210. 10.1053/j.gastro.2021.03.022 33745946 PMC8238881

[B19] Jiang P. Gu S. Pan D. Fu J. Sahu A. Hu X. (2018). Signatures of T cell dysfunction and exclusion predict cancer immunotherapy response. Nat. Med. 24, 1550–1558. 10.1038/s41591-018-0136-1 30127393 PMC6487502

[B20] Jin J. Shi Y. Zhang S. Yang S. (2020). PIK3CA mutation and clinicopathological features of colorectal cancer: A systematic review and meta-analysis. Acta Oncol. 59, 66–74. 10.1080/0284186x.2019.1664764 31545109

[B21] Kanth P. Inadomi J. M. (2021). Screening and prevention of colorectal cancer. Bmj 374, n1855. 10.1136/bmj.n1855 34526356

[B22] Lizardo D. Y. Kuang C. Hao S. Yu J. Huang Y. Zhang L. (2020). Immunotherapy efficacy on mismatch repair-deficient colorectal cancer: From bench to bedside. Biochim. Biophys. Acta. Rev. Cancer 1874, 188447. 10.1016/j.bbcan.2020.188447 33035640 PMC7886024

[B23] Luo Y. Yao Y. Wu P. Zi X. Sun N. He J. (2022). The potential role of N(7)-methylguanosine (m7G) in cancer. J. Hematol. Oncol. 15, 63. 10.1186/s13045-022-01285-5 35590385 PMC9118743

[B24] Ma J. Han H. Huang Y. Yang C. Zheng S. Cai T. (2021). METTL1/WDR4-mediated m(7)G tRNA modifications and m(7)G codon usage promote mRNA translation and lung cancer progression. Mol. Ther. 29, 3422–3435. 10.1016/j.ymthe.2021.08.005 34371184 PMC8636169

[B25] Mao X. Xu J. Wang W. Liang C. Hua J. Liu J. (2021). Crosstalk between cancer-associated fibroblasts and immune cells in the tumor microenvironment: New findings and future perspectives. Mol. Cancer 20, 131. 10.1186/s12943-021-01428-1 34635121 PMC8504100

[B26] Mei Z. B. Duan C. Y. Li C. B. Cui L. Ogino S. (2016). Prognostic role of tumor PIK3CA mutation in colorectal cancer: A systematic review and meta-analysis. Ann. Oncol. 27, 1836–1848. 10.1093/annonc/mdw264 27436848 PMC5035784

[B27] Meyer N. Penn L. Z. (2008). Reflecting on 25 years with MYC. Nat. Rev. Cancer 8, 976–990. 10.1038/nrc2231 19029958

[B28] Ming J. Wang C. (2022). N7-Methylguanosine-Related lncRNAs: Integrated analysis associated with prognosis and progression in clear cell renal cell carcinoma. Front. Genet. 13, 871899. 10.3389/fgene.2022.871899 35495133 PMC9043611

[B29] Pandolfini L. Barbieri I. Bannister A. J. Hendrick A. Andrews B. Webster N. (2019). METTL1 promotes let-7 MicroRNA processing via m7G methylation. Mol. Cell 74, 1278–1290. 10.1016/j.molcel.2019.03.040 31031083 PMC6591002

[B30] Riley R. S. June C. H. Langer R. Mitchell M. J. (2019). Delivery technologies for cancer immunotherapy. Nat. Rev. Drug Discov. 18, 175–196. 10.1038/s41573-018-0006-z 30622344 PMC6410566

[B31] Schmidt S. Gay D. Uthe F. W. Denk S. Paauwe M. Matthes N. (2019). A MYC-GCN2-eIF2α negative feedback loop limits protein synthesis to prevent MYC-dependent apoptosis in colorectal cancer. Nat. Cell Biol. 21, 1413–1424. 10.1038/s41556-019-0408-0 31685988 PMC6927814

[B32] Shi H. Chai P. Jia R. Fan X. (2020). Novel insight into the regulatory roles of diverse RNA modifications: Re-Defining the bridge between transcription and translation. Mol. Cancer 19, 78. 10.1186/s12943-020-01194-6 32303268 PMC7164178

[B33] Shi H. Wei J. He C. (2019). Where, when, and how: Context-dependent functions of RNA methylation writers, readers, and erasers. Mol. Cell 74, 640–650. 10.1016/j.molcel.2019.04.025 31100245 PMC6527355

[B34] Siegel R. L. Miller K. D. Fuchs H. E. Jemal A. (2021). Cancer statistics, 2021. Ca. Cancer J. Clin. 71, 7–33. 10.3322/caac.21654 33433946

[B35] Song P. Tayier S. Cai Z. Jia G. (2021). RNA methylation in mammalian development and cancer. Cell Biol. Toxicol. 37, 811–831. 10.1007/s10565-021-09627-8 34272618 PMC8599391

[B36] Sung H. Ferlay J. Siegel R. L. Laversanne M. Soerjomataram I. Jemal A. (2021). Global cancer statistics 2020: GLOBOCAN estimates of incidence and mortality worldwide for 36 cancers in 185 countries. Ca. Cancer J. Clin. 71, 209–249. 10.3322/caac.21660 33538338

[B37] van der Leun A. M. Thommen D. S. Schumacher T. N. (2020). CD8(+) T cell states in human cancer: Insights from single-cell analysis. Nat. Rev. Cancer 20, 218–232. 10.1038/s41568-019-0235-4 32024970 PMC7115982

[B38] Voutsadakis I. A. (2021). The landscape of PIK3CA mutations in colorectal cancer. Clin. Colorectal Cancer 20, 201–215. 10.1016/j.clcc.2021.02.003 33744168

[B39] Wei J. He C. (2021). Chromatin and transcriptional regulation by reversible RNA methylation. Curr. Opin. Cell Biol. 70, 109–115. 10.1016/j.ceb.2020.11.005 33706173 PMC8119380

[B40] Wu Q. N. Luo X. J. Liu J. Lu Y. X. Wang Y. Qi J. (2021). MYC-activated LncRNA MNX1-AS1 promotes the progression of colorectal cancer by stabilizing YB1. Cancer Res. 81, 2636–2650. 10.1158/0008-5472.Can-20-3747 33782099

[B41] Xiao J. Li W. Huang Y. Huang M. Li S. Zhai X. (2021). A next-generation sequencing-based strategy combining microsatellite instability and tumor mutation burden for comprehensive molecular diagnosis of advanced colorectal cancer. BMC Cancer 21, 282. 10.1186/s12885-021-07942-1 33726687 PMC7962287

[B42] Zhang M. Song J. Yuan W. Zhang W. Sun Z. (2021). Roles of RNA methylation on tumor immunity and clinical implications. Front. Immunol. 12, 641507. 10.3389/fimmu.2021.641507 33777035 PMC7987906

[B43] Zhang Q. Liu F. Chen W. Miao H. Liang H. Liao Z. (2021). The role of RNA m(5)C modification in cancer metastasis. Int. J. Biol. Sci. 17, 3369–3380. 10.7150/ijbs.61439 34512153 PMC8416729

[B44] Zhang Y. Zhang Z. (2020). The history and advances in cancer immunotherapy: Understanding the characteristics of tumor-infiltrating immune cells and their therapeutic implications. Cell. Mol. Immunol. 17, 807–821. 10.1038/s41423-020-0488-6 32612154 PMC7395159

