# Supplementary figures and images for: Establishment of m7G-related gene pair signature to predict overall survival in colorectal cancer
Source: Front Genet. 2022 Oct 14;13:981392. doi: 10.3389/fgene.2022.981392 (PMC9614021; doi:10.3389/fgene.2022.981392)

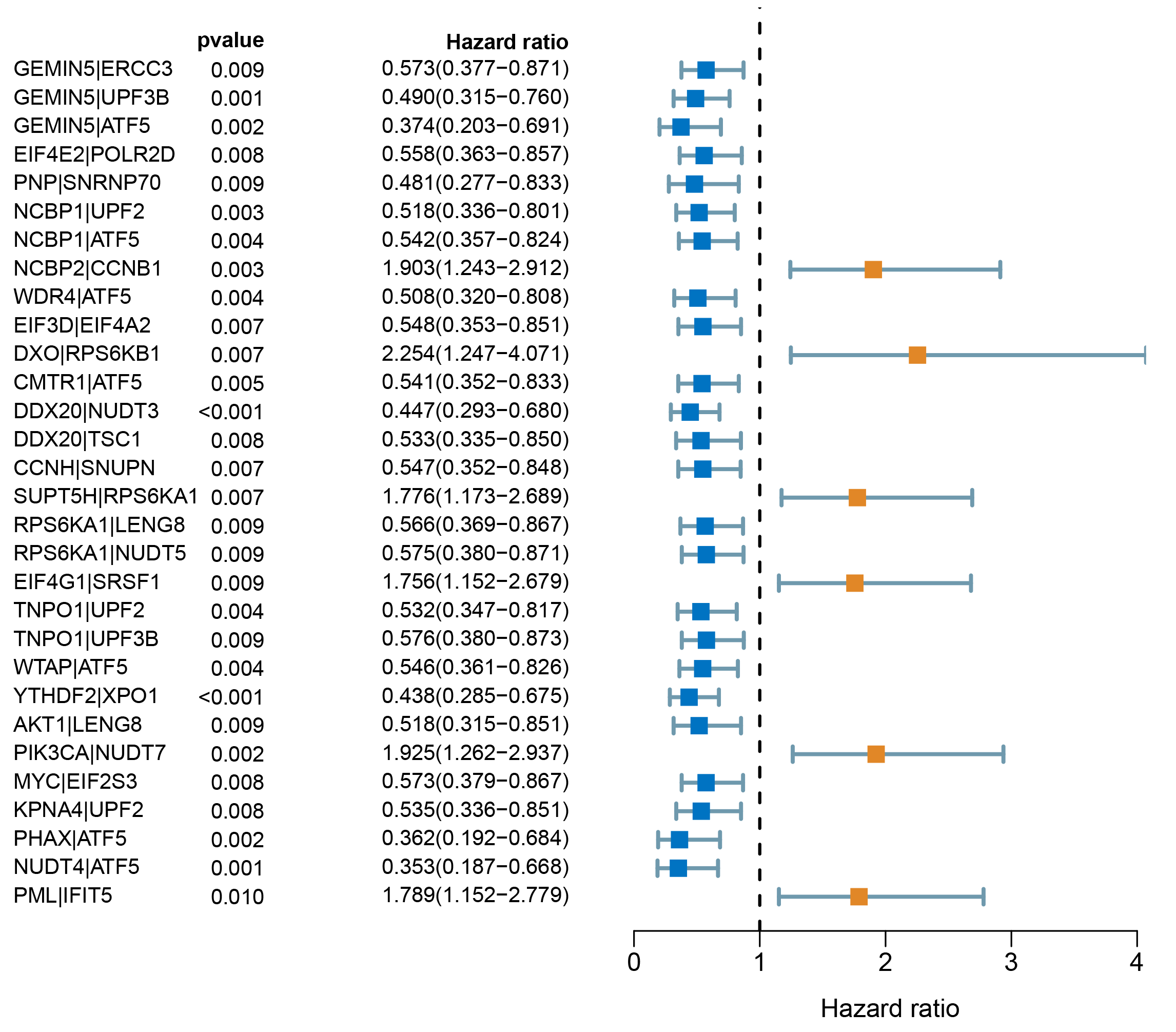

Supplement: Supplementary file 2 [file Image1.TIF]
